# Mechanism of exercise-induced analgesia: what we can learn from physically active animals

**DOI:** 10.1097/PR9.0000000000000850

**Published:** 2020-09-23

**Authors:** Joseph B. Lesnak, Kathleen A. Sluka

**Affiliations:** Department of Physical Therapy and Rehabilitation Sciences, University of Iowa, Iowa City, IA, USA

**Keywords:** Pain, Exercise, Hyperalgesia, Analgesia, Animal models, Central mechanisms, Peripheral mechanisms

## Abstract

Physical activity has become a first-line treatment in rehabilitation settings for individuals with chronic pain. However, research has only recently begun to elucidate the mechanisms of exercise-induced analgesia. Through the study of animal models, exercise has been shown to induce changes in the brain, spinal cord, immune system, and at the site of injury to prevent and reduce pain. Animal models have also explored beneficial effects of exercise through different modes of exercise including running, swimming, and resistance training. This review will discuss the central and peripheral mechanisms of exercise-induced analgesia through different modes, intensity, and duration of exercise as well as clinical applications of exercise with suggestions for future research directions.

## Abstract 2

Physical activity produces analgesia through supraspinal, spinal, and peripheral mechanisms. Currently, no exercise mode has proven to be superior to others for analgesic effects.

## 1. Introduction

Physical inactivity has become a major health concern due to its role in increasing all-cause mortality and promoting conditions such as obesity, cardiovascular disease, diabetes, cancer, dementia, and depression.^9,54,87,119,122,129,155,161^ Physical inactivity also increases the risk of developing chronic pain, with individuals with lower amounts of physical activity reporting more incidence of musculoskeletal pain.^88,89^ Furthermore, evidence-based practice guidelines recommend exercise with moderate to strong evidence for a variety of chronic pain conditions such low back pain, osteoarthritis, and fibromyalgia.^8,17,18,20,21,126^ It is recognized that an acute bout of physical activity or exercise can increase pain in individuals with chronic pain.^43,90^ Animal models have been developed to model these phenomena, show multiple underlying mechanisms, and have been reviewed elsewhere.^92,140^ Thus, although an acute bout of exercise can increase pain in individuals with chronic pain and animal models, regular physical activity and exercise can both prevent and alleviate chronic pain.

Although exercise is one of the main treatment approaches for these chronic pain conditions, there are still many questions surrounding exercise that need to be resolved. Specifically, questions still exist regarding appropriate prescription of exercise type, duration, intensity, and volume for chronic pain populations. Similarly, although exercise is typically prescribed for treatment of chronic pain, we do not fully understand the underlying mechanisms of exercise-induced pain relief. Thus, there has been a push to understand the underlying mechanisms of exercise-induced analgesia through use of animal models of exercise, which allow for a better understanding of exercise's effects on the brain, spinal cord, immune system, and periphery to produce analgesia. Elucidating these mechanisms could aid in more effective prescription and dosing of exercise for chronic pain, as well as development of new pharmacological targets for pain relief. The purpose of this review is to explore the mechanisms of exercise-induced analgesia in animal models for both pain prevention and alleviation.

## 2. Models of exercise-induced analgesia

Exercise can be performed aerobically or anaerobically and can be used with shortening (concentric), lengthening (eccentric) or static (isometric) contractions. Eccentric exercises produce muscle damage and have been used to model acute muscle pain in animals,^4,61,114,151,152^ whereas concentric-based exercises do not produce muscle damage or pain.^74,116,153^ The most commonly studied form of exercise in animal models is aerobic conditioning exercises such as treadmill running, swimming, or running wheel activity. Only one group has studied resistance training effects on pain and uses a single bout of concentric exercise.^56,58^ Thus, the majority of studies in animal models use aerobic exercise to examine mechanisms of analgesia.

Exercise-induced analgesia has been studied in multiple ways using animal models. One approach examined effects in animals by testing changes in response to painful stimuli immediately after a single exercise bout, which is comparable to studies in human subjects examining exercise-induced hypoalgesia to a single bout of exercise. This paradigm generally results in a short-lasting analgesic response (<30 minutes).^127^ More commonly, animal studies examine the effects of repeated exercise bouts over a longer period and are comparable to clinical treatment of individuals with chronic pain with a regular exercise program.^59,88,89^ These animal studies apply the repetitive exercise program either before or after a painful insult known to produce long-lasting hyperalgesia. Training sessions conducted before a painful insult explore the ability of exercise to prevent the onset of long-lasting pain, whereas training after onset of hyperalgesia will study exercises ability to alleviate pain.

The earliest work that demonstrated exercise-induced analgesia in animals was seen after short bouts of a forced cold-water swim. These studies demonstrated that swimming for as little as 3 minutes reduced pain related behaviors in response to tail shock and thermal tail-flick tests.^13–15,24,37,62,105,120,160^ Although these early studies demonstrated exercise-induced analgesia, it is hard to tease out analgesic effects due to the exercise paradigm as opposed to stress-induced analgesia by the cold water.^22^ Thus, current research has moved away from cold-water swim tests and towards less stressful exercise models. Currently, exercise models for animals use forced treadmill running, forced swimming, forced resistance training, and voluntary running wheels placed in cages. Forced treadmill running and swimming are the most commonly used exercise paradigms because they allow for control over intensity and duration of physical activity. However, these exercise models have demonstrated the ability to increase biomarkers of stress.^36^ Again, stress itself can produce analgesia through activation of opioid and serotonergic systems thus confounding results from these studies.^25,37,164^ A way to avoid this is through the use of voluntary running wheels placed in cages, which allows the animal to exercise without a stressful component. With that being said, the majority of exercise models show analgesia in pain-free animals and prevent or reverse hyperalgesia in animal models of neuropathic, inflammatory, and noninflammatory muscle pain.

**Figure 1.**
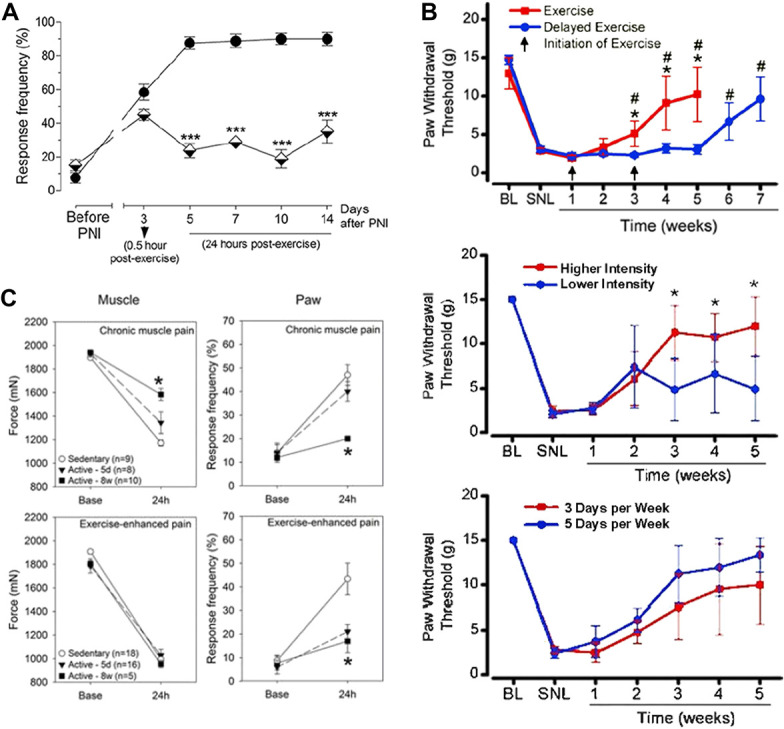
Exercise-induced analgesia. (A) Treadmill exercise after peripheral nerve injury (PNI) results in decreased mechanical hypersensitivity in as little as 2 training sessions. Filled circles represent PNI/Sedentary group and half-filled triangles represent PNI/Exercised group. Reproduced with permission from 12. (B) Exercise training resulted in reductions in hypersensitivity 2 weeks after induction of training regardless if 1 or 3 weeks passed between injury and initiation of training. Higher intensity of treadmill training resulted in a greater analgesic effect. However, no effect seen on degree of analgesia when treadmill training was conducted either 3 or 5 days a week. Reproduced with permission from 142 (C) Eight weeks of physical activity resulted in prevention of hyperalgesia both at muscle and paw after induction of noninflammatory muscle pain. Reproduced with permission from 149.

Neuropathic pain models use numerous methods to induce hyperalgesia. These models produce injury to peripheral nerves or spinal cord, or is induced by diabetes. These pain models produce both mechanical and thermal paw hyperalgesia, which last for weeks to months. Exercise protocols that start either after or before the induction of neuropathic pain show a reduction in hyperalgesia when compared to sedentary animals (Tables 1 and 2). For example, repetitive swimming protocols showed that 50, 60, or 90 minutes of swimming 5 days a week reversed both mechanical and thermal hyperalgesia after onset of training compared to sedentary animals in animal models of peripheral nerve injury.^3,28,86,150^ Similar reductions in mechanical and thermal hyperalgesia in animal models of neuropathic pain are seen in animal studies using treadmills for exercise after induction of neuropathic pain. Treadmill protocols using repeated bouts of 10, 20, 30, or 60 minutes ranging from 3 to 7 days a week have been shown to reduce hyperalgesia if started before or after the induction of nerve injury^5,10–12,26–29,31,34,35,50,63,72,73,80,81,85,96,97,101,102,131,135,149,154,156,162,165^ (Fig. 1A). Furthermore, initiation of exercise either 1 week or 3 weeks after nerve injury produced analgesia 2 weeks after initiation of training,^149^ suggesting exercise is effective regardless of when the injury occurs (Fig. 1B). Greater intensity of treadmill training (10 m/min vs 16 m/min) produced greater analgesia, whereas differences in frequency of training (3 d/wk vs 5 d/wk) did not alter analgesic effects^149^ (Fig. 1B). By contrast, treadmill training 2 weeks before injury combined with 2 weeks after injury produced the greatest analgesic effects compared with solely training either before or after injury.^11^ Interestingly, treadmill training to exhaustion during each session blocked the long-term analgesic effects of exercise and resulted in hyperalgesia that was more severe than sedentary nerve-injured animals.^35^ This suggests that intensity and volume of exercise could be a key factor in production of analgesia with higher intensity and volume producing more analgesic effects while too much intensity could prove detrimental. Finally, in exercise models where animals are trained solely before induction of neuropathic pain mixed results are seen with voluntary wheel running for 6 weeks and treadmill training for 2 weeks blunting the development of mechanical hyperalgesia,^11,65^ whereas other studies with treadmill training for 2 or 3 weeks before injury saw no effect.^131,156^ Interestingly, in experimental models where animals are given access to running wheels after induction of neuropathic pain, some studies show positive analgesic effects,^65,67,124,163^ whereas others fail to show any pain relief.^137,159^ Overall, the majority of research illustrates that various exercise models and paradigms have proven successful in preventing and alleviating neuropathic pain.

**Table 1:** Therapeutic effects of exercise.

Reference	Pain model	Animals	Exercise type	Pretraining exercise	Initiation of exercise	Exercise duration + Intensity	Effective for pain
Allen et al.^1^	OA—MIA	Male rats	Treadmill	N/A	PID 10	12–16 m/min for 30 , 4 d/wk for 5 wks	Yes for mechanical sensitivity, weightbearing symmetry, and ongoing pain
Almeida et al.^3^	Neuropathic—PSNL	Male mice	Swimming	1 min for 5 d	PID 7	10 min on days 1–3, duration increased by 10 min every 3 sessions till 50 min reached, 5 d/wk for 5 wks	Yes for mechanical and thermal sensitivity
Arbat-Plana et al.^5^	Neuropathic—SNSR	Female rats	Treadmill	N/A	PID 3	18 m/min for 60 min, 5 d/wk for 2 wks	Yes for mechanical
Bement et al.^6^	Muscle -acidic saline	Male rats	Treadmill	3.05 m/min for 5 min for 3 d	PID 0	6.1 m/min for 15 min on days 1–2, 30 min on days 3–5	Yes for muscle and mechanical sensitivity
Benson et al.^7^	EAE	Female mice	VWR	60 min access daily for 1 wk	PID 1	60 min access daily for 3 wks	Yes for mechanical sensitivity
Bobinski et al.^10^	Neuropathic—SC	Male mice	Treadmill	10 m/min for 10 min for 6 d	PID 3	10 m/min for 30 min, 5 d/wk for 2 wks	Yes for mechanical sensitivity
Bobinski et al.^11^	Neuropathic—SC	Male mice	Treadmill	10 m/min for 10 min for 6 d	PID 3	10 m/min for 30 min, 5 d/wk for 2 wks	Yes for mechanical and thermal sensitivity
Bobinski et al.^12^	Neuropathic—SC	Male mice	Treadmill	10 m/min for 10 min for 6 d	PID 3	5 d/wk for 2 wk, 30 min at 10 m/min	Yes for mechanical sensitivity
Chen et al.^28^	Neuropathic—CCI	Male rats	Treadmill or swimming	Treadmill: 20 m/min for 15 min, for 3 d swimming: N/A	1 day before injury	Graded exercise protocols, 5 d/wk for 6 wk	Both exercise protocols effective for mechanical and thermal sensitivity
Chen et al.^27^	Diabetic neuropathy	Male rats	Treadmill	N/A	Not stated	20 m/min for 60 min, 7 d/wk for 8 wks	Yes for mechanical and thermal sensitivity
Chen et al.^29^	Incision	Male rats	Treadmill	18 m/min for 15 min for 3 d	PID 8	18 m/min for 55 min, 5 d/wk for 4 wks	Yes for mechanical sensitivity
Chen et al.^30^	Incision	Male rats	Treadmill	12 m/min for 15 min for 2 d	PID 8	18 m/min for 55 min, 5 d/wk for 4 wks,	Yes for mechanical sensitivity
Chen et al.^26^	Diabetic neuropathy	Male rats	Treadmill	10 m/min for 15 min for 3 d	PID 3	Wk 1–2: 20 m/min for 30 min, wk 3–4: 25 m/min for 60 min, 7 d/wk for 4 wks	Yes for mechanical and thermal sensitivity
Chhaya et al.^31^	Neuropathic—SCI	Female rats	Treadmill	N/A	PID 5	Started at 5 m/min and increased speed till 14 m/min for 20 min, 5 d/wk for 4 wks	Yes for mechanical sensitivity
Chuganji et al.^33^	Cast	Male rats	Treadmill	N/A	PID 1	15 m/min for 30 min, 5 d/wk for 8 wks	Yes for mechanical sensitivity
Cobianchi et al.^35^	Neuropathic—CCI	Male mice	Treadmill	21 m/min till exhaustion or 60 min, 5 d/wk for 2 wks	PID 3	21 m/min till exhaustion or 60 min, 5d or 5 d/wk for 7 wks	Yes for mechanical sensitivity after 5 d of training, no for >5 d of training
Cobianchi et al.^34^	Neuropathic—SNSR	Female rats	Treadmill	N/A	PID 3	19 m/min for 60 min for 5 d	Yes for mechanical sensitivity
Cormier et al.^38^	OA—MIA	Male rats	VWR	7 or 21 d	PID 1	24 hr access 7 d/wk for 3 wks	Yes for weightbearing symmetry with 21 days prior but not 7
de Azambuja et al.^46^	Glutamate-induced pain	Male rats	Swimming	15–20 min for 6 d	PID1	30 min for 5 d	No for muscle sensitivity
Detloff et al.^50^	Neuropathic—SCI	Female rats	FWR	N/A	PID 5	14 m/min for 20 min, 5 d/wk for 5 wks	Yes for mechanical, no for thermal sensitivity
Detloff et al.^50^	Neuropathic—SCI	Female rats	FWR	N/A	PID 14 or 28	14 m/min for 20 min, 5 d/wk for 5 wks	No for mechanical sensitivity
Gong et al.^63^	Neuropathic—SNI	Male rat pups	Treadmill	N/A	PID 11	Days 1–3: 5 m/min for 10 min, days 4–6: 8 m/min for 20 min, days 8–13: 10 m/min for 30 min, days: 15–20 15 m/min for 30 min, 6 d/wk for 3 wks	Yes for mechanical sensitivity
Grace et al.^65^	Neuropathic—CCI	Male rats	VWR	N/A	PID 1 or 14	24 hr access 7 d/wk for 6 or 11 wks	Yes for mechanical sensitivity; PID 1 = PID 14
Groover et al.^67^	Diabetic neuropathy	Male mice	VWR	N/A	PID 1	24 hr access 7 d/wk for 12 wk	Yes for mechanical and visceral sensitivity
Huang et al.^72^	Neuropathic—CCI	Male rats	Treadmill	N/A	PID 8	14–16 m/min at 8% incline for 30 min, 7 d/wk for 3 wks	Yes for mechanical and thermal sensitivity
Hutchinson et al.^73^	Neuropathic—SCI	Female rats	Treadmill	12 m/min for 1 wk	PID 4	11–13 m/min for 20–25 min, 5 d/wk for 7 wks	Yes for mechanical sensitivity
Ishikawa et al.^76^	Inflammatory—kaolin + carrageenan injection	Male rats	Stimulated quad contractions	N/A	PID 1	20 min for 6 d	Yes for knee pressure pain thresholds and paw mechanical sensitivity
Kami et al.^80^	Neuropathic—CCI	Male mice	Treadmill	Wk 1: 5 d, 7 m/min for 10 min wk 2: 5 d, 7 m/min for 20–60 min	PID 2	7 m/min for 60 min for 5 d	Yes for mechanical and thermal sensitivity
Kami et al.^81^	Neuropathic—CCI	Male mice	Treadmill	Wk 1: 5 d, 7 m/min for 10 min wk 2: 5 d, 7 m/min for 20–60 min	PID 2	7 m/min for 60 min for 5 d	Yes for mechanical and thermal sensitivity
Korb et al.^85^	Neuropathic—SNSR	Male rats	Treadmill	N/A	PID 7	Wk 1: 9 m/min for 20–50 min, wk 2–4: 9 m/min for 60 min, 5 d/wk for 4 wks	Yes for mechanical sensitivity
Kuphal et al.^86^	Neuropathic—PSNL	Male rats and mice	Swimming	Rats: 90 min for 13 d mice: 30 min for 5 d	PID 1	Rats: 90 min for 25 d mice: 30 min for 6 d	Yes for thermal sensitivity for both rats and mice
Lopez-Alvarez et al.^96^	Neuropathic—SNSR	Female rats	Treadmill	19 m/min for 60 min for 1 d	PID 3	19 m/min for 60 min, 5 d/wk for 1 or 2 wks	Yes for mechanical and thermal sensitivity, 1 wk = 2 wk of training
Lopez-Alvarez et al.^97^	Neuropathic—SNSR	Female rats	Treadmill	19 m/min for 60 min for 1 d	PID 3	19 m/min for 60 min for 12 d	Yes for mechanical and thermal sensitivity
Luan et al.^101^	IDD	Male rats	Treadmill	N/A	PID 14	Wk 1: 9 m/min for 20 min, Wk 2: 11 m/min for 30 min, Wk 3–8: 13 m/min for 40 min, 7 d/wk for 1–8 wks	Yes for mechanical sensitivity
Ma et al.^103^	Diabetic neuropathy	Male rats	Treadmill	N/A	PID 1	Wk 1–2: 5 m/min at 10% incline for 10 min, wk 3–5: 10 m/min at 10% incline for 10 min, 4 d/wk for 5 wks	Yes for mechanical sensitivity
Ma et al.^102^	Diabetic neuropathy	Male rats	Treadmill	N/A	PID 1	Wk 1–2: 5 m/min at 10% incline for 10 min, wk 3–5: 10 m/min at 10% incline for 10 min, 4 d/wk for 5 wks	Yes for mechanical sensitivity
Martins et al.^106^	CRPS	Male mice	Swimming	N/A	PID 7	30 min for 5 d	Yes for mechanical sensitivity
Mifflin et al.^110^	EAE	Male and female mice	VWR	1 hr access for 3d	PID 4	1 hr access 7 d/wk until disease onset	Yes for mechanical sensitivity in females but not males
Morimoto et al.^112^	Cast	Male rats	Treadmill	12 m/min for 30 min for 3 d	PID 3	12 m/min for 30 min, 3 d/wk for 2 wks	Yes for mechanical sensitivity
Parent-Vachon et al.^124^	Neuropathic—SNI	Female mice	VWR	N/A	PID 14	24 hr access 7 d/wk for 6 wks	Yes for mechanical sensitivity
Pitcher et al.^125^	Inflammatory—CFA	Male rats	VWR	N/A	PID 3	2 hr/d access 4 d/wk for 3 wks	Yes for weightbearing asymmetry and thermal sensitivity
Ross et al.^128^	I/R	Male mice	Treadmill	N/A	PID 0	13 m/min for 45 min for 1 d	No for mechanical sensitivity
Safakhah et al.^131^	Neuropathic—CCI	Male rats	Treadmill	10 m/min for 10 min for 5 d	PID 5	16 m/min for 30 min, 5 d/wk for 3 wks	Yes for mechanical and thermal sensitivity
Sanford et al.^132^	Chronic stress	Female rats	VWR	N/A	PID 1	24 hr access 6 d/wk for 3 wks	Yes for visceral sensitivity
Shankarappa et al.^135^	Diabetic neuropathy	Male rats	Treadmill	18 m/min for 60 min for 5 d	PID 1	18 m/min for 60 min, 5 d/wk for 10 wks	Yes for mechanical sensitivity
Sharma et al.^136^	Muscle -acidic saline	Female mice	Treadmill	13 m/min for 3d (no duration given)	PID 5	Wk 1: 13 m/min for 30 min, Wk 2: 14 m/min for 40 min, Wk 3: 15–16 m/min for 45 min, 5 d/wk for 3 wks	Yes for muscle and mechanical sensitivity
Sheahan et al.^137^	Neuropathic—SNI	Male mice	VWR	N/A	PID 8–10	2 or 12 hr access, 5-6 d/wk 2 wks	No for mechanical sensitivity
Smith et al.^146^	Overuse muscle injury	Female rats	Treadmill	N/A	Initiated during wk 5 of overuse injury model	23 m/min for 60 min, 5 d/wk for 6 wks	No for mechanical sensitivity
Stagg et al.^149^	Neuropathic—SNL	Male rats	Treadmill	18 m/min for 10 min, 2 d/wk for 2 wks	PID 7 or 21	14–16 m/min for 30 min, 3 or 5 d/wk for 5 wks; OR 10 or 16 m/min for 30 min, 5 d/wk for 5 wks	Yes for mechanical and thermal sensitivity; PID 7 = 21; 3 d/wk = 5 d/wk; 16 m/min: yes, 10 m/min: no
Sun et al.^150^	TNT	Male rats	Swimming	N/A	PID 7	Wk 1: 10–50 min, Wk 2–5: 60 min, 5 d/wk for 5 wks,	Yes for mechanical sensitivity
Tsai et al.^154^	Neuropathic—CCI	Male rats	Treadmill	N/A	PID 6	14–16 m/min for 30 min at 0 or 8% incline, 7 d/wk for 3 wks	Yes for mechanical and thermal sensitivity 8% > 0% incline
Wakaizumi et al.^156^	Neuropathic—PSNL	Male mice	Treadmill	None or 6 or 12 m/min for 60 min 5 d/wk for 2 wks	PID 1	6 or 12 m/min for 60 min 5 d/wk for 1 or 2 wks,	Yes for mechanical and thermal sensitivity (6 m/min > 12 m/min)
Whitehead et al.^159^	Neuropathic—CCI	Male rats	VWR	60 min access for 1 wk	PID 2–3	60 min access for 7 or 18 d	No for mechanical sensitivity
Yamaoka et al.^162^	Neuropathic—PSNL	Female rats	Treadmill	N/A	PID 1	20 m/min at 10° incline for 10 min, 5 d/wk 6 wks	Yes for mechanical and thermal sensitivity
Ye et al.^163^	Neuropathic—ART	Male mice	VWR	N/A	PID 0	2 hr access 5 d/wk for 13 wks	Yes for mechanical and thermal sensitivity
Yoon et al.^165^	Diabetic neuropathy	Male rats	Treadmill	5 m/min for 20 min for 2 d	PID 7	10 m/min for 60 min 5 d/wk for 6 wks	Yes for mechanical sensitivity

Table for effects of exercise on pain when exercise is initiated after induction of pain model.

ART, antiretroviral therapy; CCI, chronic constriction injury; CFA, complete Freund's adjuvant; CPRS, complex regional pain syndrome; EAE, experimental autoimmune encephalomyelitis; FWR, forced wheel running; I/R, ischemia and reperfusion; IDD, intervertebral disk degeneration; m/min, meters per min; MIA, monosodium iodoacetate; N/A, not applicable; OA, osteoarthritis; PID, postinjury day; PSNL, peripheral sciatic nerve ligation; SC, sciatic crush; SCI, spinal cord injury; SNI, spared nerve injury; SNSR, sciatic nerve resection and repair; TNT, tibial neuroma transposition; VWR, voluntary wheel running.

**Table 2:** Preventative effects of exercise.

Reference	Pain model	Animals	Exercise type	Exercise Duration + Intensity	Duration of pretraining	Effective for pain
Bobinski et al.^11^	Neuropathic –SC	Male mice	Treadmill	10 m/min for 30 min, 5 d/wk	2 wk	Yes for mechanical, no for thermal sensitivity
Brito et al.^16^	Muscle -acidic saline	Male and female mice	VWR	24 hr access 7 d/wk	8 wk	Yes for muscle and mechanical sensitivity
de Azambaja et al.^45^	Inflammatory-carrageenan	Male rats	Swimming	40 min, 5 d/wk, 4% BW attached to animals' body	10 wk	Yes for muscle sensitivity
Grace et al.^65^	Neuropathic—CCI	Male rats	VWR	24 hr access 7 d/wk	6 wk	Yes for mechanical sensitivity
Kuphal et al.^86^	Inflammatory-formalin	Male rats	Swimming	90 min	9 d	No for phase 1 pain response, yes for phase 2 pain response
Leung et al.^91^	Muscle—activity-induced	Male and female mice	VWR	24 hr access 7 d/wk	8 wk	Yes for muscle and mechanical sensitivity
Lima et al.^92^	Muscle—activity-induced	Male and female mice	VWR	24 hr access	5 d	Yes for mechanical, no for muscle sensitivity
Martins et al.^107^	Glutamate-induced pain	Male mice	Swimming	30 min, 5 d/wk	1–2 wk	Yes for mechanical sensitivity 2 wk > 1 wk
Ross et al.^128^	Muscle—I/R	Male mice	VWR	24 hr access	2 d	Yes for mechanical sensitivity
Sabharwal et al.^130^	Muscle—activity-induced	Male and female mice	VWR	24 hr access 7 d/wk	5 d or 8 wk	5 d: yes for mechanical, no for muscle sensitivity; 8 wk: yes for mechanical and muscle sensitivity
Safakhah et al.^131^	Neuropathic—CCI	Male rats	Treadmill	16 m/min for 30 min 5 d/wk	3 wk	No for mechanical and thermal sensitivity
Sheahan et al.^137^	Inflammatory-formalin	Male mice	VWR	2 or 12 hr/d access, 5–6 d/wk	1–4 wk	No for nocifensive behaviors
Sluka et al.^142^	Muscle—acidic saline or activity-induced	Male mice	VWR	24 hr access 7 d/wk	5 d or 8 wk	5 d: yes for mechanical, no for muscle sensitivity; 8 wk: yes for mechanical and muscle sensitivity
Wakaizumi et al.^156^	Neuropathic—PSNL	Male mice	Treadmill	6 or 12 m/min for 60 min, 5 d/wk	2 wk	No for mechanical or thermal sensitivity

Table for effects of exercise on pain when exercise is performed before induction of pain model.

CCI, chronic constriction injury; I/R, ischemia and reperfusion; m/min, meters per min; PSNL, peripheral sciatic nerve ligation; SC, sciatic crush; VWR, voluntary wheel running.

In models of noninflammatory muscle pain, hyperalgesia is induced through a dual stimulus model of acid injections into the gastrocnemius muscle, which are administered 5 days apart. This pain model results in long-lasting widespread hyperalgesia without tissue damage.^111,141^ In this model, exercise produces analgesia when performed as a preventative tool before muscle insult or as a therapeutic tool when started after the muscle insult (Tables 1 and 2). Development of mechanical hyperalgesia measured at the paw, ie, secondary hyperalgesia, is completely blocked in animals granted access to running wheels for 5 days or 8 weeks before induction of model.^93,130,142^ Further blockade of muscle hyperalgesia, ie, primary hyperalgesia, is only seen in animals granted access to running wheels for 8 weeks before induction of the model suggesting longer duration of training is needed to block primary hyperalgesia than secondary hyperalgesia^16,91,130^ (Fig. 1C). It is worth noting that distance run by animals with free access to running wheels is variable ranging between 0.2 and 5 km/d; yet, there is no correlation between distance run and degree of analgesia.^65,91,142^ These data suggest that there is a minimal threshold of activity necessary to protect against development of hyperalgesia. Similarly, treadmill training starting after induction of muscle pain caused a transient reversal of muscle pain immediately after the training session, whereas 3 weeks of training resulted in a complete reversal of the hyperalgesia.^6,136^ Thus, exercise can be effective for producing analgesic effects in models of noninflammatory muscle pain.

Exercise has also shown to be beneficial for pain relief in less widely studied pain models including osteoarthritis,^1,38^ incisional pain model,^29,30^ inflammatory pain model,^45,76,86,125^ complex regional pain syndrome,^106^ intervertebral disk degeneration (IDD),^101^ immobilization-induced pain,^33,112^ chronic stress-induced pain,^132^ ischemia and reperfusion,^128^ glutamate-induced pain,^107^ and experimental autoimmune encephalomyelitis^7,110^ (Tables 1 and 2). Although behavioral data are important for demonstrating the analgesic effects of exercise, understanding the mechanisms through which physical activity is producing these effects will be beneficial for future development of therapeutic targets for pain reduction. Consequently, the rest of this review covers the central, peripheral, and neuroimmune changes that occur to produce exercise-induced analgesia.

**Figure 2.**
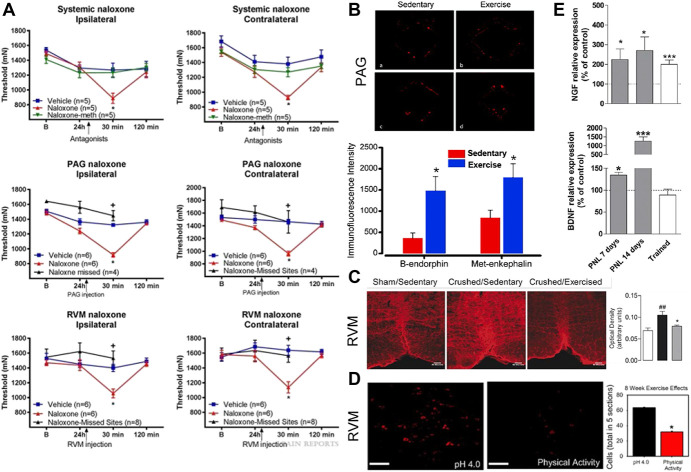
Central mechanisms of exercise-induced analgesia. (A) Systemic naloxone but not naloxone methiodide reversed analgesic effects of exercise. Naloxone delivered directly into periaqueductal gray (PAG) or rostroventromedial medulla (RVM) also reversed the analgesic effects of exercise. Reproduced with permission from 16. (B) Treadmill training for 5 weeks resulted in increases in beta endorphin and met-enkephalin in PAG. Reproduced with permission from 149. (C) Two weeks of treadmill training reversed the increases in SERT in RVM seen after neuropathic injury. Reproduced with permission from 10. (D) Eight weeks of physical activity blocked increases in p-NR1 in RVM after induction of noninflammatory muscle pain. Reproduced with permission from 142. (E) Swimming reduced neuropathic injury increases in NGF and BDNF in lumbar DRG. Reproduced with permission from 3. BDNF, brain-derived neurotrophic factor; NGF, nerve growth factor.

## 3. Central mechanism of exercise-induced analgesia

### 3.1. Endogenous opioids

One of the most widely studied central mechanisms of exercise-induced analgesia is the role of endogenous opioids in producing the analgesic effects. Early work showed that in healthy animals, systemic administration of naloxone, an opioid receptor antagonist, blocks the acute analgesic effects of short bouts of swimming.^13,24,62,120,137,160^ More recently, these behavioral effects have been replicated in exercise models of treadmill running, voluntary wheel running and resistance training as well as in pain models of noninflammatory muscle pain, neuropathic pain, arthritic pain, and glutamate-induced pain.^1,6,16,47,58,93,106,107,147^ Specifically, 8 weeks of voluntary running wheel activity blocked the production of hyperalgesia in a model of noninflammatory muscle pain. Naloxone administered systemically or directly into the periaqueductal gray (PAG) or the rostral ventral medial medulla (RVM), 2 brain locations important in endogenous opioid system, blocked these analgesic effects, whereas systemic injection of naloxone methiodide, a peripherally acting opioid antagonist, had no effect suggesting the central but not peripheral effects of exercise on mediating the endogenous opioid system^16^ (Fig. 2A). Similarly, the analgesic effects of treadmill running for neuropathic pain were reversed with either subcutaneous or intracerebroventricular injection of naloxone but not naloxone methiodide.^149^ Also, treadmill training after induction of knee osteoarthritis caused decrease in tactile sensitivity, improvement in weightbearing symmetry, and reduction of ongoing pain, and these analgesic exercise effects were reversed with systemic naloxone.^1^ Finally, 5 days of voluntary running wheel activity prevented the development of hyperalgesia in an activity-induced muscle pain model in wild-type but not mu-opioid receptor knockout mice demonstrating exercise produces its analgesic effects through activation of mu-opioid receptors.^93^

Studies have also shown changes in the endogenous opioid system due to exercise in several locations. Increases in beta-endorphin and met-enkephalin, 2 endogenous opioid peptides, were found in both the PAG and RVM after 5 weeks of treadmill running and in the PAG and hypothalamus after 8 weeks of treadmill training, whereas increases in beta-endorphin in cerebrospinal fluid were found to be elevated for up to 48 hours after a single exercise bout^33,70,149^ (Fig. 2B). Also, increased opioid peptides met-enkephalin, leu-enkephalin, and beta-endorphin were found in frontal cortex of female rats given access to running wheels for 4 weeks after nerve injury.^124^ Similarly, increases in enkephalin were found in lumbar dorsal root ganglia (DRG) after 6 weeks of treadmill training.^165^ Finally, both forced and voluntary running activity for 7 days increased mu-opioid receptor expression in the hippocampus. Interestingly, when animals were subjected to forced or voluntary running activity for 45 sessions over a 9-week span, the increases in mu-opioid receptor were absent.^47^ This suggests that repeated activation of the opioid system by exercise can produce a compensatory downregulation of opioid receptor expression, similar to that seen in chronic administration of exogenous opioids such as morphine.^2^ In parallel, behavior studies show that chronic, but not acute, running wheel exposure in uninjured animals decreases analgesic effects of morphine, a mu-opioid agonist, and U50,488H, a kappa opioid agonist, and increases in withdrawal-like symptoms after administration of opioid receptor antagonists.^40,82,143^ Thus, current research suggests that exercise-induced analgesia is mediated by endogenous opioid activity.

### 3.2. Serotonergic system

The serotonergic system has been widely studied for its role in both the development and alleviation of chronic pain. Not surprisingly, exercise has been shown to produce its analgesic effects through modulation of the serotonergic system. Serotonin (5-HT) is elevated in the brainstem after a single 60-minute bout of swimming exercise and in the dorsal horn of the lumbar spinal cord after 15 minutes of treadmill running in pain-free rats.^51,60^ When rats swam 6 days a week for 4 weeks, increased levels of 5-HT were found in both the brainstem and parieto-occipital cortex and these increased levels were still significantly higher than sedentary controls one week after cessation of exercise.^51^ Similarly, in pain-free animals, increased levels of 5-HT were found in the cerebellum and midbrain after 8 weeks of treadmill training, whereas increased levels of 5-HT immunoreactivity were found in RVM after 4 weeks of treadmill training.^19,85^ Interestingly, after peripheral nerve injury, increased levels of 5-HT immunoreactivity were found bilaterally in the lumbosacral ventral horn of the spinal cord after 4 weeks of treadmill training but not in animals that underwent sham nerve injury.^85^ Also, animals with the peripheral nerve injury did not see increases in 5-HT immunoreactivity in the RVM, suggesting exercise could potentially produce different central nervous system effects depending on injury state of animal.^85^

In the RVM, increases in serotonin are analgesic with extracellular levels of serotonin controlled by the serotonin transporter (SERT).^75,95^ The SERT is increased, whereas 5-HT is decreased, in the RVM and brainstem after induction of neuropathic pain.^10^ Two weeks of treadmill training after induction of neuropathic pain reversed the increases in SERT and decreases in 5-HT with a corresponding reduction in mechanical hyperalgesia^10^ (Fig. 2C). Animals pretreated with ρ-chlorophenylalanine, a 5-HT synthesis inhibitor, blocked brainstem increases of 5-HT and prevented the analgesic effect of treadmill exercise in animals with neuropathic pain, repeated stimulated muscle contractions in pain-free animals, and 5 days of swimming exercise before acetic acid pain.^10,69,107^ Similar to the neuropathic pain model, there are increases in SERT in the RVM after induction of a noninflammatory muscle pain model, which is prevented by 8 weeks of prior voluntary running wheel activity.^16^ Finally, after induction of the noninflammatory pain model, blockade of SERT in the RVM, by microinjection of the selective serotonin reuptake inhibitor fluoxetine, reversed hyperalgesia showing modulation of SERT alters pain behaviors.^16,93^ Together, these data suggest that exercise-induced release of serotonin as well as changes in central serotonergic system is critical to the analgesic effects of regular exercise.

Recent evidence has also suggested an interactive role between the opioid and serotonergic systems to produce exercise-induced analgesia. Serotonin neurons in the RVM receive inputs from PAG opioid neurons and blockade of serotonin receptors in the PAG or spinal cord reduces the analgesic effects of morphine.^25,83,134^ Increases in SERT occur in the RVM after induction of an activity-induced pain model and 5 days of voluntary wheel running before induction of the model can block the SERT increases.^93^ However, physically active animals pretreated with systemic naloxone show greater SERT immunoreactivity in the RVM compared with physically active animals receiving vehicle. Similarly, physically active mu-opioid receptor knockout mice showed increased SERT immunoreactivity in RVM compared with physically active wild-type animals. Together, these data suggest that activation of opioid receptors during exercise is crucial for reducing expression of SERT in the brainstem to promote exercise-induced analgesia.

### 3.3. Endocannabinoids

Activation of the endocannabinoid system has also been attributed to the analgesic effects of exercise. Endocannabinoid receptors are located in the PAG, RVM, and dorsal horn of the spinal cord, all of which are locations known to be important for pain modulation.^71,109,157^ There are 2 naturally occurring ligands, anandamide (AEA) and 2-arachidonylglycerol (2-AG), that bind to the endocannabinoid CB_1_ and CB_2_ receptors to produce analgesic effects. In both human and animal studies, acute bouts of exercise increase circulating levels of endocannabinoids, which contributes to the feeling of “runner's high” associated with exercise.^39,55–57,84,148^ Endocannabinoid-mediated exercise-induced analgesia has been found after exercise in both healthy animals and animals subjected to glutamate-induced pain.^56,57,107^ In healthy animals, a single bout of treadmill training or resistance training increased nociceptive threshold to mechanical stimuli, which was blocked by pretreatment with systemic, intrathecal, or intracerebroventricular injections of CB_1_ and CB_2_ inverse agonists, AM251 and AM630, suggesting the endocannabinoid system is working at multiple locations to produce exercise-induced analgesia.^56,57^ Also, the antinociceptive response could be prolonged through systemic, intrathecal, or intracerebroventricular pretreatment with endocannabinoid metabolizing enzyme inhibitors. Similarly, 30 minutes of swimming for 1 week before intraplantar glutamate injection produced an antinociceptive effect, which was blocked by pretreatment with intrathecal, but not intraplantar, administration of a CB_1_ antagonist (AM281).^107^ Finally, increased expression of CB_1_ receptors were found in the dorsolateral and ventrolateral PAG after a single bout of treadmill training or resistance training and injection of AM251 into the PAG before exercise reduced this upregulation.^56,57^ Interestingly, similar to the serotonin-opioid interaction for producing analgesia, evidence has suggested a similar relationship between the endocannabinoid and opioid systems^23,39,115^; however, this relationship has not been studied in animal models of exercise-induced analgesia. Thus, evidence suggests there are supraspinal and spinal actions of exercise-induced analgesia involving the endocannabinoid system.

### 3.4. NDMA receptor alterations

The *N*-methyl-d-aspartate (NMDA) receptor is an excitatory glutamate receptor which has been implicated in the development of hyperalgesia. Increases in phosphorylation of the NR1 subunit (p-NR1) of the NMDA receptor in the RVM are crucial for the development of chronic muscle hyperalgesia. In models of noninflammatory and activity-induced muscle pain, there is increased p-NR1 expression in the RVM, and downregulation or pharmacological blockade of NR1 in the RVM prevents development of hyperalgesia^41,42,139^ showing a role for NMDA receptors in chronic muscle pain. More recent work shows that physical activity blocks the increases in p-NR1 in the RVM after muscle injury^142^ (Fig. 2D). Similarly, an hour of running daily for a week prevented increases in p-NR1 expression in the superficial dorsal horn in a model of experimental autoimmune encephalomyelitis.^7^ Finally, treadmill training was able to reduce increased NR1 expression in the lumbar spinal cord resulting from an incisional pain model.^29^ This suggests that exercise reduces excitatory transmission by preventing phosphorylation of the NMDA receptors in the central nervous system.

### 3.5. Noradrenergic system

The noradrenergic system also plays a role in the analgesic effects of exercise. Receptors for catecholamines (α1, α2, β2-adrenergic receptors) are found in locations known to mediate pain such as the PAG, locus coeruleus, dorsal raphe and spinal cord DRG.^117^ The noradrenergic system is activated during exercise to increase release of catecholamines.^52^ Early studies show that short bouts of swimming produced analgesia is potentiated by systemic pretreatment with norepinephrine, clonidine (α2-adrenergic agonist), or desipramine (norepinephrine reuptake inhibitor) when compared to swimming alone, whereas systemic or intracerebroventricular pretreatment with α1-or α2-adrenergic receptor antagonists prevented the swim-induced analgesia.^14,15,121^ Similarly, systemic pretreatment with α2, α2-A, or α2-C adrenergic receptor antagonists blocked exercise-induced analgesia from a single bout of either treadmill training or resistance training; there was no effect when administered intrathecally or intracerebroventricularly.^48^ Furthermore, in α2-A/C adrenergic receptor knockout mice, treadmill training failed to produce any antinociceptive effects to swimming or treadmill training.^48^ Finally, after induction of a neuropathic pain model, regular treadmill training prevented the downregulation α1 and β2 receptors in the dorsal horn of the spinal cord. Exercise was also shown to upregulate α1 and β2 receptors in the locus coeruleus and dorsal raphe. Systemic inhibition of the β2 receptors with butoxamine before exercise training completely blocked the analgesic effects of exercise. Furthermore, neuropathic injury-induced increases in activated microglia in the locus coeruleus were reduced with exercise; however, this effect was blocked by butoxamine treatment.^97^ This work demonstrates the role of the noradrenergic system in producing exercises' analgesic effects. Future work will need to be done to further determine location of action of the noradrenergic system in the production of exercise-induced analgesia.

### 3.6. Role of adenosine receptors

There has been a small amount of research into the role of adenosine and its receptor subtypes, A1 and A2A, in the role of exercise-induced analgesia. One study found swimming exercise for one week before intraplantar glutamate injection prevented glutamate-induced nociception.^107^ When these animals were pretreated with an A1 adenosine receptor antagonist, 1,3-dipropyl-8-cyclopentylxanthine (DPCPX), either intrathecally or intraplantarly, the antinociception effects of the swimming exercise was prevented. Similarly, swimming exercise mitigated mechanical hyperalgesia in an animal model of complex regional pain syndrome, but systemic pretreatment with caffeine (adenosine receptor antagonist) or an A1 receptor antagonist (DPCPX), but not an A2A receptor antagonist (ZM241385), blocked these analgesic effects.^106^ Furthermore, systemic pretreatment with an adenosine degradation inhibitor (erythro-9-(2-hydroxy-3nonyl) adenine; EHNA) potentiated exercise-induced analgesia. Together, these data suggest that exercise produces analgesia through A1 adenosine receptors both centrally and peripherally.

### 3.7. Changes in spinal cord and dorsal root ganglia

There have been multiple studies that have examined a number of different neurotransmitters, intracellular messengers, transcription factors, and growth factors in the spinal cord or DRG in a variety of animal models of exercise-induced analgesia. Neuropathic injury increases the intracellular messenger PLCγ-1 and the transcription factor CREB in the dorsal horn of the spinal cord, and increases both nerve growth factor (NGF) and brain-derived neurotrophic factor in spinal cord and DRG.^3^ Regular swimming (5 days/wk for 5 weeks) or 2 weeks of treadmill running not only prevented the development of hyperalgesia, but also returned all these measures (PLCg-1, CREB, NGF, and brain-derived neurotrophic factor) to normal uninjured levels^3,12^ (Fig. 2E). Similarly, 2 weeks of swimming prevented the increased intracellular messenger phosphorylated-PKA in the lumbar spinal cord after intraplantar glutamate injection,^107^ a single bout of treadmill training blocked the increase in c-FOS expression in the spinal cord after intraplantar formalin injection,^94^ and 3 weeks of treadmill training before induction of paw incisional pain model reduced the amount of phosphorylated-p38 in the spinal cord.^64^ Also, in a model of knee osteoarthritis there was an increase in calcitonin gene-related peptide in the dorsal horn of the lumbar spinal cord in laminae I–VI. Six days of electrically stimulated quadriceps contractions starting at onset of knee osteoarthritis completely blocked this increase.^76^ In addition, in models of diabetic neuropathy, there are increases in TRPV1 channels, phosphorylated-p38, phosphorylated m-TOR and its downstream signals S6K1 and 4E-BP1, and inflammatory cytokines IL-1β, IL-6, and TNFα in lumbar DRG.^102,165^ Regular treadmill training starting at the onset of diabetes was able to block the increases in TRPV1 channels, intracellular messengers, and inflammatory cytokines. Finally, neuropathic injury decreases the inhibitory neurotransmitter gamma-aminobutyric acid (GABA) and glutamic acid decarboxylase (which converts glutamate to GABA) in the dorsal horn of the spinal cord, which is prevented by regular treadmill running 60 minutes per day 5 days a week.^80^ Thus, this research suggests that exercise works to reverse increases in pronociceptive intracellular messengers, transcription factors, and growth factors while restoring antinociceptive mediators in the spinal cord.

Electrophysiology studies have found increased excitability of spinal cord dorsal horn neurons after injection of NGF into the multifidus muscles over 5 days. Swimming for 5 days between NGF injections blocked increases in measures of neuronal excitability in the spinal cord.^46^ Namely, the exercise blocked increases in the number of neurons responding to deep tissue stimulation, the receptive field size, and the resting activity compared to sedentary animals. There are also increases in DRG neurons excitability after induction of neuropathic pain with increased resting membrane potential, promoted spike frequency and spike counts, and decreased threshold potential in lumbar DRG.^163^ Voluntary running wheel activity for 2 hours a day, 5 days a week, during model induction completely blocked all increases in DRG excitability measures. Finally, in a pain model of diabetic peripheral neuropathy, treadmill training for 60 minutes, 5 days a week, delayed the onset of mechanical hypersensitivity. In sedentary animals, a >2.5 increase in calcium channel current density was measured at lumbar DRG.^135^ In animals that exercised, this increase in calcium channel current density was completely blocked. This research suggests that exercise is also working at the level of the spinal cord and DRG to produce its analgesic effects through altering electrophysiological changes that occur due to injury.

## 4. Neuroimmune mechanisms of exercise-induced analgesia

**Figure 3.**
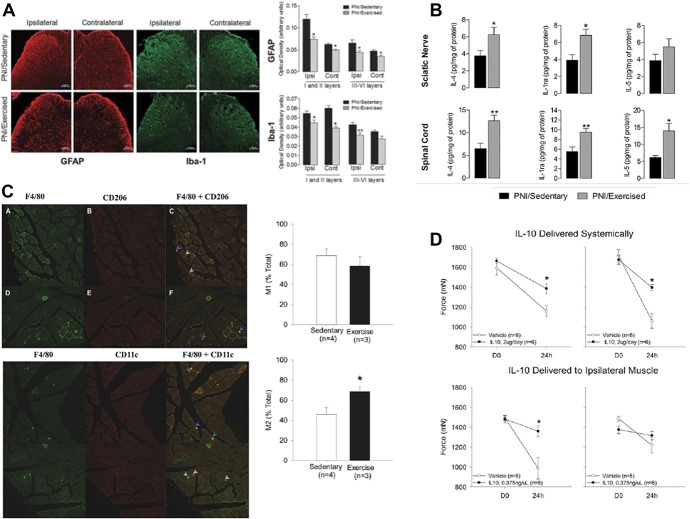
Immune system mechanisms of exercise-induced analgesia. (A) Treadmill training for 2 weeks after neuropathic injury reduced hyperactive astrocytes (GFAP) and microglia (Iba-1) in lumbar spinal cord dorsal horns. Reproduced with permission from 12. (B) Treadmill training after sciatic nerve injury increased anti-inflammatory cytokines in spinal cord and sciatic nerve. Reproduced with permission from 12. (C) Physical activity increased percentage of M2 macrophages in gastrocnemius muscle (F4/80 macrophage stain, CD206 M2 macrophage stain, CD11c M1 macrophage stain). Reproduced with permission from 91. (D) Pretreatment with IL-10 blocked hyperalgesia in response to noninflammatory muscle pain. Reproduced with permission from 91.

Alterations in the immune system by exercise have been studied both in the central nervous system and locally at the site of injury. Centrally, immune responses are modulated through activity of microglial cells and astrocytes, which can alter their morphology resulting in production of different levels of pro-inflammatory and anti-inflammatory cytokines. Specifically, microglia and astrocytes in the spinal cord will transform into a hypertrophic state after sciatic nerve injury resulting in an activated state that will increase pro-inflammatory cytokine production.^3,7,12,31,35,63,65,81^ In parallel, increases in pro-inflammatory cytokines TNF-α, IL-1β, and IL-6 and decreases in anti-inflammatory cytokines IL-4, IL-5, and IL-1ra have been reported in the RVM, spinal cord, and DRG after induction of sciatic nerve injury.^10–12,63,65,165^ Spinal cord increases in hyperactive microglia and astrocytes are blocked by treadmill running or swimming both before and after sciatic nerve injury^3,7,12,31,35,65,81^ (Fig. 3A). Furthermore, animals that exercised had reduced expression of pro-inflammatory cytokines (TNF-α, IL-1β, and IL-6) and increases in anti-inflammatory cytokines (IL-4, IL-1ra, IL-5, and IL-10)^10–12,63,65,165^ (Fig. 3B). Finally, in IL-4 knockout mice or mice given an IL-4 antibody during treadmill training, exercise-induced analgesia in response to a sciatic nerve injury was completely blocked, suggesting that the increases in anti-inflammatory cytokines are part of the mechanism through which exercise protects and reverses the development of hyperalgesia.^12^

Exercise also modulates the immune response in the periphery at the site of injury. A model of knee osteoarthritis found increased total number of macrophages in the synovium of the arthritic knee. The increase in macrophages was attenuated by electrically stimulated quadriceps contractions starting at the onset of knee osteoarthritis.^76^ Also, treadmill training blocked the increases in pro-inflammatory cytokines (TNF-α, IL-1β, and IL-6), increases in chemokine CCL2,^65^ and decreases in anti-inflammatory cytokines (IL-4, IL1ra, and IL-10) that are found in the sciatic nerve after induction of neuropathic pain.^11,12,26,28,72,154^ Furthermore, sciatic nerve injury was found to alter macrophage distribution in the sciatic nerve. Macrophages are immune cells that have different phenotypes depending on their cytokine release profile: M1 macrophages release pro-inflammatory cytokines, whereas M2 macrophages release anti-inflammatory cytokines.^113^ There is an increased proportion of M1 to M2 macrophages in the sciatic nerve after injury.^12,65^ Regular treadmill training before or after injury increased the number of M2 vs M1 macrophages, increased serum IL-10, and decreased IL-1β and CCL2 in the serum and sciatic nerve.^12,65,72^ Similarly, physically active, uninjured mice show an increased proportion of M2 macrophages in the muscle^91^ (Fig. 3C). In a noninflammatory muscle pain model, the analgesia produced by 8 weeks of running wheel activity is prevented by systemic or intramuscular blockade of IL-10 receptors, whereas administration of IL-10 systemically or into the muscle mimicked the analgesic effects similar to exercise^91^ (Fig. 3D). Also, in an ischemic model of muscle pain, there was an increase in IL-1β levels in the muscle which was blocked with prior exercise.^128^ Furthermore, blockade of IL-1 receptors prevented the exercise-induced analgesia, whereas treatment with IL-1 resulted in decreases in pain behaviors.^128^ Finally, in a pain model of IDD, there was an increase in IL-1β, TNFα, and leptin in the multifidus muscle, all of which were decreased by voluntary running wheel activity.^77^ Thus, this research suggests that exercise can alter macrophage phenotype distribution and subsequent pro-inflammatory and anti-inflammatory cytokine profiles in the periphery at the site of injury to promote analgesia.

## 5. Peripheral mechanisms of exercise-induced analgesia

There have been a few studies on exercise-induced changes in the periphery that can lead to exercise's analgesic effects. In the paw, after sciatic nerve injury, there are increases in nerve fiber sprouting from adjacent areas into the denervated areas resulting in mechanical hyperalgesia both at the medial and lateral test sites of the paw.^96^ Treadmill running for 60 minutes a day decreased the amount of nerve sprouting and reinnervation of plantar skin from the medial paw into the denervated lateral paw resulting in decreased responses to mechanical stimuli. Also, tibial nerve injury resulted in increasing plasma levels of leptin and decreased levels of adiponectin with a subsequent mechanical hyperalgesia.^150^ Swimming exercise 5 days a week reversed mechanical hyperalgesia and normalized circulating leptin and adiponectin levels. Systemic administration of leptin after swimming sessions blocked the analgesic effects of exercise, whereas administration of adiponectin alone was able to decrease mechanical hyperalgesia. Finally, a model of IDD results in degenerative changes in the nucleus pulposus and anulus fibrosus. Daily treadmill training initiated after onset of the IDD model resulted in increased cell counts in the nucleus pulposus and anulus fibrosus, as well as increased markers of cell proliferation in the intervertebral disk and the adjacent epiphyseal cartilage when compared to sedentary controls.^101^ Thus, these data suggest that alterations in the periphery at or away from the site of injury can also impact pain behaviors and exercise can help to mitigate any pronociceptive changes at these sites.

## 6. Clinical implications

From a clinical standpoint, this research on animal models of exercise-induced analgesia could provide the basis for new clinical studies to improve exercise prescription guidelines for humans. First, exercise works to both prevent onset of pain and resolve pain after injury. Currently, 58/64 (90%) articles published on the topic of exercise-induced analgesia demonstrate positive effects of exercise in as little as one bout of activity. Also, this research demonstrates that specificity of exercise might not be as important as the act of exercise itself. Exercise-induced analgesia is demonstrated in exercise models of running, swimming, and resistance training. Similarly, in human studies, different types of exercise programs have been applied to chronic pain patients with no difference in outcomes seen between the different exercise programs.^68,79,133^ Also, both aerobic and resistance training exercise programs are listed as exercise recommendations for low back pain, osteoarthritis, and fibromyalgia.^8,17,18,20,21,126^ This lack of specificity of exercise is possibly a result of the multitude of mechanisms through which exercise can reduce pain. Therefore, exercise prescription should be dependent upon patient preferences, therapist training, available equipment, cost, and safety.^133^ Furthermore, there is evidence to suggest that duration and intensity of exercise can impact the degree of exercise-induced analgesia. Higher amounts of duration and intensity resulted in a greater degree of analgesia; however, too much intensity was found to have detrimental effects, suggesting a possible inverted U-shaped curve when it comes to exercise intensity and pain relief.^35,94,149^ Thus, dosing of exercise needs to be done appropriately to maximize pain relief while limiting negative side effects. Finally, as previously noted individuals with chronic pain may fail to receive pain relief from an acute bout of exercise or may suffer from exercise-induced pain.^127^ This increase in pain with an acute bout exercise could be a barrier to exercise adherence. This can be managed through appropriate dosing of exercise to decrease exercise-induced symptom flares, or through treatments that reduce activity-induced pain. Our recent clinical trial shows that transcutaneous electrical nerve stimulation (TENS) significantly reduces activity-induced pain when compared to placebo or no-TENS in women with fibromyalgia,^44^ suggesting TENS could be an effective treatment to manage activity-induced pain. Another approach could be to use psychological and educational interventions to decrease fear-avoidance behavior surrounding activity and catastrophic thinking regarding exercise-induced hyperalgesia.^104,118,127^

## 7. Future directions

Although work has repeatedly shown the analgesic effects of exercise, the underlying mechanism for this analgesia has only recently begun to be elucidated. Continued research into the mechanisms through which exercise can prevent and reduce pain will help develop intervention targets for treating chronic pain. Specifically, research into the mechanisms through which exercise prevents pain vs reduces pain could reveal different mechanisms. Also, we do not know if the neurological and immune response to exercise is the same in pain-free animals as compared to animals after injury. Human studies have suggested that overweight individuals see greater increases in pro-inflammatory cytokines (IL-6, IL-8, and TNFα) compared with lean individuals after acute bouts of exercise.^32^ Thus, future work needs to study the impact of comorbidity factors on the analgesic mechanisms of exercise. Although studies have shown that all modes of exercise are beneficial, a majority of the research in animal models has been on aerobic-based exercise (running and swimming) as opposed to strength training which is typically performed in rehabilitation settings. Although all exercise modes have beneficial effects, we do not know if aerobic and resistance training-based exercise programs are producing analgesia through the same mechanisms. Similarly, we do not know the adequate amount of dose or minimal effective dose for these exercise programs. Thus, further research needs to be conducted on other forms of exercise as well as on different dosing of exercise to improve exercise prescription in chronic pain patients. Finally, nearly all exercise-induced analgesia research has been conducted in populations using only male animals. Recent research has called for inclusion of female animals in all experiments due to sexual differences found in hyperalgesia mechanisms and pain phenotypes.^66,78,98–100,123,145–147,158^ Because mechanistic differences in pain development have been seen between the sexes, we cannot assume the same mechanistic effects of exercise-induced analgesia between males and females. Of the research that has used both sexes while studying exercise-induced analgesia, mixed results have been found, with some finding sexual differences in exercise-induced analgesia^110^, whereas others have not.^93,139^ Therefore, future studies need to include both sexes in their experiments to determine if there are possible sex differences in the mechanisms of exercise-induced analgesia. The incorporation of females into animal studies is also crucial because chronic pain conditions are more prevalent in women.^53^ By only including male animals, the generalizability to the population of individuals who suffer from chronic pain conditions is limited. All of this research would help improve utilization, adherence, and prescription of exercise for chronic pain patients.

## Disclosures

The authors have no conflicts of interest to declare.
